# Do professionalism, leadership, and resilience combine for professional identity formation? Evidence from confirmatory factor analysis

**DOI:** 10.3389/fmed.2024.1385489

**Published:** 2024-06-13

**Authors:** Aine Ryan, Catherine N. Moran, David Byrne, Anne Hickey, Fiona Boland, Denis W. Harkin, Shaista S. Guraya, Abdelsalam Bensaaud, Frank Doyle

**Affiliations:** ^1^Centre for Professionalism in Medicine and Health Sciences at Royal College of Surgeons in Ireland, University of Medicine and Health Sciences, Dublin, Ireland; ^2^Department of Health Psychology, School of Population Health, Royal College of Surgeons in Ireland University of Medicine and Health Sciences, Dublin, Ireland; ^3^Data Science Centre, School of Population Health, Royal College of Surgeons in Ireland University of Medicine and Health Sciences, Dublin, Ireland; ^4^Institute of Learning, Mohammad Bin Rashid University, Dubai, United Arab Emirates

**Keywords:** medical education research, professional identity formation, professionalism, leadership, resilience, confirmatory factor analysis

## Abstract

**Introduction:**

Professional identity formation (PIF) is an ongoing, self-reflective process involving habits of thinking, feeling and acting like a physician and is an integral component of medical education. While qualitative work has suggested that PIF is informed by professionalism, resilience, and leadership, there is a dearth of quantitative work in this area. Multiple methods build rigor and the present study aimed to quantitatively assess the relative psychometric contributions of professionalism, resilience, and leadership constructs to informing PIF, using a latent factor analysis approach.

**Methods:**

We analyzed data from the PILLAR study, which is an online cross-sectional assessment of a pre-clinical cohort of medical students in the RCSI University of Medicine and Health Sciences, Dublin, using established and validated quantitative measures in each area of interest: PIF, professionalism, leadership and resilience. A total of 76 items, combining four validated scales, along with a selection of demographic questions, were used. The hypothesis that PIF is informed by, and correlates with, professionalism, resilience and leadership was examined by conducting a confirmatory factor analysis of a proposed three-factor higher-order model. Model estimation used Maximum Likelihood Method (MLM) with geomin rotation. The hypothesized (measurement) model was examined against an alternative (saturated) model, as well as a three-factor model.

**Results:**

Latent variable analysis from 1,311 students demonstrated that a three-factor higher-order model best fit the data; suggesting PIF is informed by professionalism, resilience, and leadership, and that these constructs are statistically distinct and account for differential aspects of PIF. This higher-order model of PIF outperformed both the saturated model and the three-factor model. The analysis of which component may be the most or least influential was inconclusive, and the overall model was not influenced by year of training.

**Discussion:**

Building upon existing conceptual contentions, our study is the first to quantitatively support the contribution of professionalism, resilience, and leadership to the development of professional identity, and to delineate the inter-relationships between PIF and these constructs. This information can be used by medical educators when designing curricula and educational strategies intended to enhance PIF. Future work should seek to assess the influence of these constructs longitudinally.

## Introduction

It has been proposed that professional identity formation (PIF) should be a central focus of medical education and this recommendation has been supported by several researchers (1–3). Professional identity formation can be defined as “*A representation of self, achieved in stages over time during which the characteristics, values, and norms of the medical profession are internalized, resulting in an individual thinking, acting, and feeling like a physician*” (1). University programs can have a significant impact on PIF, in that they are important contributors to the formation of professional identity in medical students (4–6). It has been suggested that the PIF of future physicians is strongly influenced by PIF curricula and pedagogic strategies (7). Curricula should provide adequate opportunity for a student to reconsider their own values and beliefs, by relating to the behaviors that are expected by the medical profession, colleagues, and patients (8). Strategies such as incorporating process-based outcomes along with guided self-assessment have been suggested as potential effective methods to integrate PIF into curricula (9). In terms of educational strategies, several methods have been reported to be effective in PIF. Namely, the importance of role models and mentors, early exposure to patients and the clinical environment, structured feedback, and narrative reflection (10–14). Experiences during clinical teaching have been identified as being particularly important contributors to the student’s formation of their professional identity (15). Similarly the importance of mentorship and clinical placement experience has been proposed as an important factors in fostering resilience and professionalism and leadership skills in medical students (16–18). It has been reported that during longitudinal integrated clerkships, where students participate in the comprehensive care of patients over time, the responsibility students assume as they become co-providers of patient care contributes significantly to their professional identity through the development of meaningful interpersonal relationships along with belonging to a community of practice (19). It has also been suggested if there a lack of professional identity development during training it could result in a loss of trained students from their respective industry (20). The concept of PIF in medicine is not new (21) and much of the existing literature concentrates on what PIF is and why it is important (22). PIF is considered to be of equal importance to the acquisition of clinical knowledge and skills (23). Furthermore, there is an ample amount of research outlining how PIF is developed and is based on multiple theoretical perspectives including developmental and social psychology theory (1–3, 7, 12, 24).

The concept of PIF is complex, multi-factorial, and has been identified to have multiple domains (11, 25). There has been some valid debate in the literature about the difficulty of objectively assessing PIF due to it being a private individual experience (26), however, it has been suggested that if PIF is to be a significant educational objective, robust assessment or markers of its development should be established (23, 27). Furthermore, a systematic review of the literature concluded that there is a dearth of studies which examine the psychometric validity of professional identity measures suggesting that further psychometric validation of quantitative PIF assessments, or its subcomponents, is warranted (5).

In the area of medical education professionalism has been demonstrated to be a key contributor to PIF (11). Traditionally, a summative assessment of professional behaviors has been used as a surrogate for PIF (27). This may be assessed through the completion of professionalism surveys and reflective assignments (28, 29). However, emotional resilience and leadership have also been identified as key elements in forming a personal and professional identity (23, 25, 29, 30). For PIF to occur, medical educators should have a clear understanding of the influences which impact this important developmental process. While multiple other constructs have also been argued to comprise PIF, we deliberately focused on professionalism, leadership and resilience as they are important components in our University’s new Personal and Professional Identity curriculum and investigation into their assessment was required. While literature has linked these factors conceptually and theoretically, it has done so in a qualitative manner, with, to the best of our knowledge, no evidence from quantitative studies. Applying psychometric scaling analysis techniques could allow researchers to delineate quantitatively the interrelationships, if any, among these various constructs, providing support or new insights on these theoretical contentions. Indeed, identifying these interrelationships and the degree to which they may influence PIF could provide significant information for the development of educational strategies for PIF. Our group has recently proposed a quantitative progress assessment of PIF, including professionalism, leadership and resilience (22), and a latent variable analysis of this dataset, therefore, provides a unique opportunity to ascertain whether these constructs do indeed contribute to PIF development. Specifically, we can ascertain the most appropriate theoretical model of PIF, for example comparing models where PIF is informed by resilience, leadership and professionalism (a higher-order model), or alternatives where these constructs are separate (a three-factor model), or if these simply indicate an overall single dimension (a saturated model).

## Aim

The present study aimed to quantitatively investigate the extent to which PIF is informed by professionalism, leadership, and resilience, using a latent factor analysis approach.

## Methods

### Study design

The present study reports a quantitative, psychometric evaluation of the factorial structure of PIF in medical students using data obtained from cross-sectional data from the broader PILLAR (Professional Identity Formation, ProfessionaLism, Leadership, And Resilience) study [for further detail see reference (23)]. PILLAR is an online assessment of the entire cohort of pre-clinical medical students at the RCSI University of Medicine and Health Sciences, Dublin, Ireland.^1^ A comprehensive online assessment of PIF, professionalism, leadership, and resilience was conducted using a 76-item questionnaire, which comprised four validated scales and additional questions on participant demographics. All items from the original PILLAR assessment were included in this study’s analysis. The PILLAR assessment was embedded as a compulsory part of the professionalism curriculum in pre-clinical year modules. Although compulsory, students provided voluntary, explicit, and written informed consent if they agreed to their data being used for research purposes. Ethical approval was obtained from the RCSI Research Ethics Committee before conducting the study (REC202005016). The methodology adheres to the STROBE reporting guidelines (31).

### Setting

Assessments were completed online by pre-clinical medical students at RCSI University of Medicine and Health Sciences, Dublin, Ireland.

### Participants

All 1,427 pre-clinical medical students were invited to complete the PILLAR assessment between September 2020 and February 2021, see our methods paper for further information on methodology (23). The recruitment sample comprised all students from Foundation Year (pre-medicine; FY), Years 1–3 of direct-entry undergraduate medicine (DEM), and Years 1–2 of graduate-entry medicine (GEM). Of this sample, 1,311 participants (93% of eligible medical students) consented to their data being used as part of the research.

### Variables

The PILLAR assessment comprised four valid and standardized scales examining PIF, professionalism, resilience, and leadership (23).

#### Professional identity formation

PIF was assessed by the 9-item Professional Self-Identity Questionnaire (PSIQ) (32). The PSIQ measures 9 domains of professional self-identity within health and social care professions including teamwork, communication, patient or client assessment, cultural awareness, ethical awareness, using patient or client records, dealing with emergencies, reflective practice, and teaching. Respondents rated each statement as to how they would currently identify themselves when undertaking each professional activity on a scale from 0 to 6, where 0 represents the first day as a student doctor, and 6 indicates a newly qualified doctor. After the study pilot, an additional item relating to PIF was included in the study protocol; namely, “I feel like a member of the medical profession.” Participants rated this item along a 5-point response scale from “Strongly disagree” to “Strongly agree.”

#### Professionalism

Professionalism was captured by 25 items from a 27-item measure of perceptions among medical students toward unprofessional behaviors (33). Two items about inappropriate dress (“Women’s dress,” “Men’s dress”) were excluded as they were not deemed suitable for the present cohort. For each statement, participants responded “yes/no” as to whether they observed, participated in, or considered the behavior to be unprofessional. To simplify analysis as per previous research, and to observe parsimony in the attitudinal assessment of professionalism, only the “perception of professionalism” responses were included in the factor analysis (34).

#### Leadership

The Medical Leadership Competency Framework (35) outlines the leadership competencies expected of practicing clinicians. Participants considered 15 items on personal qualities and working with others and rated each item as 0 “Very little/None of the time,” 1 “Some of the time,” or 2 “A lot of the time.” Scores were averaged for each domain, where higher scores were indicative of greater levels of leadership.

#### Resilience

Resilience was measured by the Brief Resilience Scale, which comprises six statement items on the perceived ability to cope with stress (36). Items were rated along a 5-point Likert scale from “Strongly Disagree” to “Strongly Agree,” with higher scores indicating higher levels of perceived resilience.

### Data sources

The study investigators provided brief presentations to each year of medical students to introduce the PILLAR assessment and invite them to participate in the research study component. The assessment was distributed to students via online SurveyMonkey software, coordinated by the RCSI Quality Enhancement Office (QEO) which acted as an independent data controller. During class time, the QEO sent automated, individualized emails to all eligible students with a link to complete the assessment. Students were also provided with two weekly reminders. The link took participants to an online participant information leaflet and consent form where students either consented or declined for their information to be used for research purposes. The QEO completely anonymized the responses for consenting participants, removing all identifiable data, before transferring the data to the research team.

### Bias

We addressed potential non-response, selection, sampling, and attrition bias through our methodology, by having PILLAR as a compulsory assessment for pre-clinical students, thereby ascertaining data from all relevant pre-clinical years. Due to issues with curriculum reform, it was not possible to have PILLAR as a compulsory assessment for the clinical years, although this will be addressed in the future.

### Study size

During September 2020 and February 2021, all pre-clinical students completed the assessment and were invited to participate in this study (*n* = 1,427).

### Statistical methods

The hypothesis that PIF is informed by professionalism (Pro), resilience (Res) and leadership (Lead) was examined by conducting a confirmatory factor analysis of a three-factor higher-order model. The hypothesized (measurement) model was examined against two alternatives: a saturated model, which posited that all professionalism, leadership and resilience items loaded directly onto a single PIF construct, as well as a three-factor model, which assessed professionalism, leadership and resilience as three independent constructs that did not load onto a common PIF factor. Analyses were conducted using R (v4.1.2) in R Studio (v2022.02.0, Build 443) (37). Uni-and multivariate normality tests were conducted using MVN (v5.9) (38). Confirmatory factor analysis was conducted using Lavaan (v.06–9), Psych (v2.1.9) and semTools (v0.5–5) packages (39–41). Model estimation used the Maximum Likelihood Method (MLM) with geomin rotation. Reliability was determined as McDonald’s Omega using MBESS (v4.9.2) (42). Data were found to violate the assumption of multivariate normality (see Supplementary Appendix 1), which can result in inflated *χ*^2^-values, subsequently causing inflated model fit indices. To insulate *χ*^2^ against the deviation from multivariate normality, and to produce robust standard errors and significance values, the robust Maximum Likelihood Method (MLR) was used when estimating model parameters (43). Goodness-of-fit was examined using absolute fit indices [Root Mean Square Error of Approximation (RMSEA) and Standardized Root Mean Square Residual (SRMR)], which are considered to be acceptable at <0.8 and < 0.06, respectively (44) and relative fit indices [Close Fit Index (CFI) and Tucker-Lewis Index (TLI)], which are considered to be acceptable at >0.90 (45). Inspection of modification indices (MIs) suggested that model fit could be improved if the error covariance for a number of items was constraint-free. Specifically, several items on the Professionalism factor were indicated. The suggested unconstrained error covariances were introduced one by one until the remaining MI values were below the 3.84 threshold (46). It was considered that each of the three factors would make relatively independent contributions to the construct of PIF. To assess this, discriminant validity was examined in several ways. First, the average variance extracted (AVE) for each factor was compared to the average shared variance (ASV) among the three factors (47). Then, the square root of the AVE for each factor was examined against respective standardized correlation coefficients with other factors. Multi-group modeling was then used to assess the equivalence of the measurement model across the first three academic years of undergraduate study. Analysis of variance (ANOVA) was used to compare a multi-group configural model, calculating independent factor loadings and intercepts for each year, with a metric invariant model (where factor loadings were fixed across years) and a scalar invariant model (where factor loadings and intercepts were fixed across years). Critical change thresholds were as follows; RMSEA >0.015, SRMR >0.010, CFI/TLI > 0.010 (48).

## Results

### Participants

A total of 1,427 students were invited to complete the PILLAR assessment, with *n* = 1,331 (93%) students responding and *n* = 1,311 (92%) consenting to their data being used for research. The mean age was 22 ± 3.0 years with just over half of the participants being female. In terms of country of origin, the majority of students were from four individual regions/countries, the Middle East (29%), North America (21.3%), Ireland (16.5%), and Malaysia (11.5%), reflecting the diversity of the student population. Further details on descriptive statistics are in our original paper (23).

### Outcome data

#### Main results: factor analysis—hypothesized model

**Figure 1:**
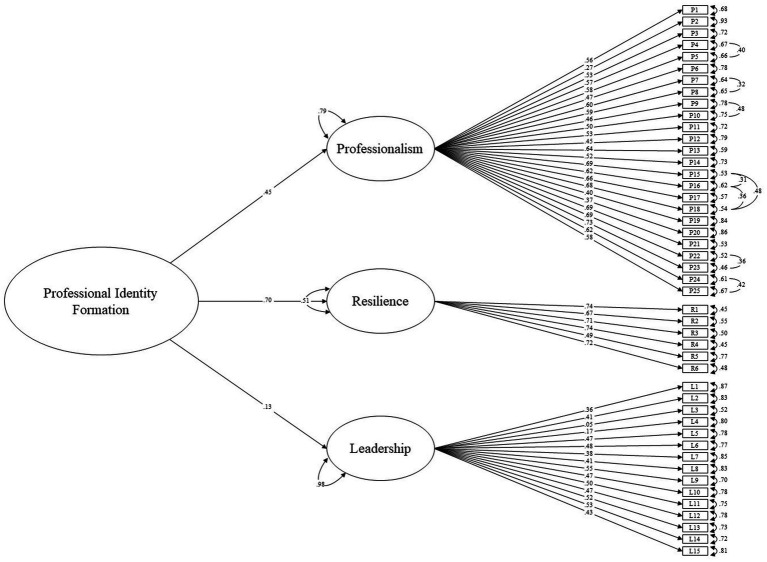
Higher order model of professional identity formation.

The hypothesized measurement model was estimated [*χ*^2^(986) = 3896.34, *p* < 0.001]. By the aforementioned criteria, absolute fit indices demonstrated acceptable fit: RMSEA (0.052, 90% CI = 0.050–0.054), SRMR (0.050), but relative fit indices were outside of acceptable values: CFI (0.81), TLI (0.80). Leadership demonstrated the largest factor loading onto PIF (0.70), followed by resilience (0.54), then professionalism (0.27). The items that were then constraint freed and their respective bivariate correlation coefficients are presented in Table 1. The revised model was significant [*χ*^2^(971) = 2482.09, *p* < 0.001] and demonstrated improved fit across most indices, with relative fit indices now presenting with acceptable values: RMSEA (0.038, 90% CI = 0.036–0.040), SRMR (0.042), CFI (0.90), TLI (0.90). Reliability analysis using McDonald’s Omega (**ω**) indicated that each of the three factors was reliable and that factor reliability could not be improved by removing items: professionalism **ω** = 0.92 (95% CI = 0.920–0.932), resilience **ω** = 0.83 (95% CI = 0.814–0.842), leadership **ω** = 0.79 (95% CI = 0.774–0.807). The model schematic and parameter estimates, including path coefficients and error terms, can be seen in Figure 1, while standardized factor loadings, Omega coefficients and average variance extracted (AVE) can be seen in Table 2. Contrary to the unmodified model, Resilience demonstrated the largest factor loading onto PIF (0.70), followed by professionalism (0.45), and then leadership (0.13). Discriminant validity was observed in relation to the three factors, as the AVE was greater than the ASV (47) and the square root of the AVE was greater than the correlation coefficients for all factors (Table 3) (49).

**Table 1:** Professionalism item covariance and correlations (> 0.07).

Items					Covariance	*r*
P9	drugevent	~~	P10	penrep	0.48	0.73
P24	feedbakctoNCHDs	~~	P25	feedbackfromNCHDsCons	0.42	0.82
P4	scrubstimeoff	~~	P5	scrubsoutsidehosp	0.40	0.76
P22	consenwosupervision	~~	P23	procedurebeyondlevel	0.36	0.89
P16	discussptspublic	~~	P18	derogatorycomment	0.36	0.85
P7	mistakenfordoctor	~~	P8	introducedasdoctor	0.32	0.77
P15	takefoodpatients	~~	P18	derogatorycomment	0.36	0.87
P15	takefoodpatients	~~	P16	discussptspublic	0.31	0.84

**Table 2:** Descriptive statistics, item factor loadings, alpha coefficients, and AVE values.

Item #	Item	Mean	SD	Skew	Kurtosis	Std. loading	Omega	AVE
Professionalism							0.92	0.35
P1	latetorounds	1.58	0.83	−1.42	0.05	0.56		
P2	abslectures	1.84	0.43	−2.84	7.56	0.25		
P3	workrooms	1.48	0.78	−1.05	−0.55	0.51		
P4	scrubstimeoff	1.17	0.88	−0.35	−1.63	0.58		
P5	scrubsoutsidehosp	1.30	0.83	−0.62	−1.26	0.59		
P6	funpatients	1.88	0.43	−3.61	11.85	0.46		
P7	mistakedr	1.58	0.76	−1.40	0.18	0.61		
P8	introdr	1.34	0.87	−0.72	−1.30	0.60		
P9	drugevent	0.84	0.83	0.31	−1.49	0.47		
P10	penrep	0.81	0.69	0.27	−0.90	0.51		
P11	impairedcolleague	1.12	0.87	−0.24	−1.65	0.53		
P12	whitecoat	1.64	0.69	−1.64	1.08	0.44		
P13	eatcorridors	1.48	0.80	−1.07	−0.60	0.64		
P14	takefoodlecture	1.57	0.73	−1.40	0.24	0.51		
P15	takefoodpatients	1.71	0.68	−2.04	2.29	0.71		
P16	discussptspublic	1.76	0.63	−2.30	3.50	0.64		
P17	personalconversat	1.30	0.79	.-59	−1.18	0.65		
P18	derogatorycomment	1.73	0.67	−2.14	2.70	0.70		
P19	intoxevents	1.55	0.71	−1.27	0.12	0.39		
P20	destructcompetitive	1.75	0.58	−2.19	3.46	0.36		
P21	discussbeyondlevel	1.52	0.81	−1.20	−0.40	0.69		
P22	consenwosupervision	1.41	0.88	−0.90	−1.11	0.71		
P23	procedurebeyondlevel	1.58	0.80	−1.43	0.97	0.76		
P24	feedbakctoNCHDs	1.16	0.95	−0.32	−1.82	0.64		
P25	feedbackfromNCHDsCons	1.06	0.96	−0.13	−1.90	0.59		
Resilience							0.83	0.47
R1	bounceback	3.52	1.02	−0.51	−0.40	0.75		
R2	throughstressevent	3.09	1.04	−0.15	−0.85	0.66		
R3	recoverstressevent	3.36	1.02	−0.41	−0.54	0.71		
R4	snapback	3.25	0.10	−0.31	−0.73	0.74		
R5	throughdifficulttimes	3.13	0.99	−0.15	−0.68	0.48		
R6	timeoversetback	3.35	1.05	−0.40	−0.56	0.72		
Leadership							0.79	0.20
L1	ownvaluesprinciples	1.63	0.54	−1.10	0.17	0.35		
L2	seekfeedback	1.26	0.67	−0.35	−0.70	0.40		
L3	remaincalm	1.40	0.61	−0.47	−0.65	0.33		
L4	delivercommitments	1.38	0.60	−0.40	−0.68	0.37		
L5	seeopportunities	1.52	0.56	−0.62	−0.65	0.49		
L6	applymylearning	1.43	0.61	−0.61	−0.57	0.50		
L7	actopenhonest	1.89	0.33	−2.49	7.97	0.38		
L8	speakoutcompromised	1.43	0.59	−0.49	−0.66	0.40		
L9	identifyoppcolab	1.44	0.61	−0.62	−0.56	0.54		
L10	shareinfo	1.58	0.58	−1.01	−0.03	0.46		
L11	communicateclear	0.16	0.56	−0.76	−0.45	0.51		
L12	listenfeelingsothers	1.80	0.42	−1.77	1.94	0.46		
L13	seekcontribution	1.48	0.60	−0.72	−0.45	0.50		
L14	mxconflictinterest	1.51	0.59	−0.77	−0.38	0.53		
L15	acknowledgeffort	1.80	0.41	−1.84	2.18	0.42		

**Table 3:** Standardized scale correlation coefficients.

	Professionalism	Resilience	Leadership
Professionalism	1.00		
Resilience	0.24	1.00	
Leadership	0.31	0.50	1.00

#### Comparison of hypothesized models to alternatives

The hypothesized three-dimensional factor structure of PIF was then compared with an alternative unidimensional (saturated) model using modified indices as per the measurement model, whereby professionalism, resilience and leadership items were allowed to load onto a single PIF factor. A Chi-square difference test was conducted, which indicated that the hypothesized measurement model was a better fit than the alternative model at a significance level of *p* < 0.001. This was further evidenced when comparing the measurement model fit indices to those of the alternative model 1. The measurement model was also compared against an alternative three-factor model whereby professionalism, resilience and leadership items loaded onto respective factors, but these factors did not load onto a higher-order PIF factor. A Chi-square difference test again indicated that the measurement model presented with a better fit than did alternative model 2 at a significance level of *p* < 0.001. This was also reflected in the fit indices. The outcomes of the comparisons of the measurement model with the alternative models (see Table 4) provide further support for the hypothesis that professionalism, resilience and leadership are statistically distinct constructs and that these constructs inform a higher-order construct of professional identity formation.

**Table 4:** Model fit statistics.

	RMSEA 90%CI		
	DF	AIC	BIC	Chisq	Chisq diff	DF diff	*p*	SRMR	RMSEA	Lower	Upper	CFI	TLI
Measurement model	976	104622.55	105159.94	2822.27				0.042	0.038	0.036	0.040	0.90	0.90
Alternative 1: Saturated	981	109547.12	110059.92	7756.85	2448.10	5	<0.001	0.097	0.074	0.072	0.076	0.61	0.59
Alternative 2: 3-factor	978	104884.08	105411.24	3087.81	258.09	2	<0.001	0.046	0.040	0.039	0.042	0.88	0.87

#### Model equivalence across academic year

Multi-group modeling was then conducted to assess the stability of the higher order 3-factor model of PIF over time. Initially, a comparison of fit indices for a configural model with those of both a metric and scalar model suggested little difference in fit across models. This was further assessed using ANOVA, which indicated non-invariance in relation to both the PIF model (metric invariance) and item scoring (scalar invariance) across the three academic years (see Table 5).

**Table 5:** Invariance analysis by academic year.

	RMSEA 90%CI		
	DF	AIC	BIC	Chisq	Chisq diff	DF diff	*p*	SRMR	RMSEA	Lower	Upper	CFI	TLI
Configural model	2,928	74189.39	77362.35	4718.99				0.059	0.041	0.038	0.043	0.88	0.88
Metric invariance	3,022	75136.63	76858.69	4854.23	126.83	94	<0.05	0.064	0.040	0.038	0.043	0.88	0.88
Scalar invariance	3,106	75153.85	76472.98	5039.45	364.11	84	<0.001	0.065	0.042	0.039	0.044	0.87	0.87

## Discussion

This paper is the first to conduct and compare factor analytic models of PIF and its contributors. The main findings are that a three-factor higher-order model is the best fit for the data, suggesting that PIF is informed by professionalism, resilience and leadership, which are statistically distinct and account for different aspects of PIF. This higher-order model of PIF outperforms both the saturated model (where all items are allowed to load onto one component) and the three-factor model (where items load on their respective factors, but the factors do not load onto PIF).

Our results provide important quantitative support for the previous qualitative work that suggested PIF is positively influenced by emotional resilience and leadership (25, 29). These results have practical implications for medical education in that they substantiate incorporating professionalism, leadership and resilience into curricula and educational interventions which aim to specifically support PIF. Initially, leadership was found to be the most influential component of PIF, with professionalism being the least influential. However, the professionalism scale required several index modifications to achieve a statistically (while theoretically) sound higher-order PIF model. In the resulting revised measurement model, resilience is presented as the most influential component of PIF, while leadership is presented as the least influential, which are novel and potentially important findings, if replicated. While the models are theoretically identical, the revised measurement model is more statistically sound, as the collinearity issue with the professionalism measure is addressed and the model fit is improved. In light of the required modifications of professionalism and its subsequent impact on PIF loadings, inferences regarding which component of PIF might be most or least influential should be considered inconclusive for now. Future research could aim to explore the respective influences of professionalism, resilience and leadership on PIF with an alternative measure of perceptions of professionalism.

The results of invariance analyses indicated that both the higher-order model of PIF and participants’ perceptions of PIF may change over time. The relatively small difference in Chi-square and fit indices between the configural and metric models suggests that it may be possible to obtain a PIF model that is invariant across the three academic years. Therefore, formal curricular input may be needed to modify these perceptions. Considering the performance of the professionalism scale, it may be best to further examine this with an alternative measure of perceptions of professionalism. As students progress through their undergraduate studies, it is plausible that curricular input and environmental factors might augment perceptions of PIF, or that hidden curricula may indeed subvert or otherwise confound efforts to promote PIF over time (50). This could inform differences in item scores across academic years, resulting in the noted scalar invariance (51).

Although there were some issues with the professionalism measure, the results of this analysis support the viability of a 3-factor higher-order model of PIF, comprising professionalism, resilience and leadership. If these results are replicated in the future, it would compound the suggestion that investing in training in each of these aspects would be core to appropriate PIF formation in future physicians.

### Limitations and future research

The major limitation of this paper is the lack of data from students in their clinical years. This will be addressed in future work, as the PILLAR assessment is longitudinal and future cohorts will continue to complete it into the clinical years in a reformed curriculum. The data are cross-sectional only, and future longitudinal work may show different results. Our regression analysis demonstrated significant differences among years on certain variables, however, there was no clear evidence of increasing or decreasing scores by year. Our results point to potential cohort effects and that each class should be taken on its own merits, rather than necessarily expecting a gradient of results from cross-sectional data. However, this result may point to a lack of sensitivity to change in these instruments, which contrasts with other work on the use of essays which do show change over time (52). Future iterations of PILLAR, with longitudinal data for each student, will shed light on these issues. Another potential limitation can be noted in relation to the professionalism measure. The loading for professionalism was initially small. Inspection of modification indices suggested that the overall model fit could be improved by freeing several error covariances. The terms that were freed all pertained to the professionalism factor, which subsequently increased its loading onto PIF. However, this had a detrimental effect on the loading for leadership. In light of what appears to be a significant collinearity issue across several pairs of items on the professionalism scale, it may be that this measure is inadequate. In this regard, it is also instructive to consider that modifying these indices may have affected invariances outcomes (53), thus our conclusions about the relative contributions of each factor to PIF are tentative. Although the higher-order model of PIF as constituting professionalism, resilience and leadership still demonstrated adequate fit, further research could usefully explore the potential for a more optimal and parsimonious higher-order PIF model using an alternative measure of perceptions of professionalism [e.g., the Professionalism Assessment Scale (PAS)] that may not require modified indices (54).

Additionally, while the sample obtained can be considered representative of undergraduate medical students (covering 3 years and two entry pathways), findings might not reflect other types of students, or indeed other types of professions. Further research could be undertaken to examine if the 3-factor higher-order model of PIF might be evident among different types of undergraduate students, for example, those studying engineering, accounting or computer science. It may also be useful to explore if the higher-order model is found among students of other universities. Research could also examine if this model remains stable as students enter into medical professions, and could explore other professions, such as consultancy, law or fast-moving consumer goods.

To conclude, our study is the first to quantitatively support the contribution of professionalism, resilience, and leadership to the development of professional identity, and to delineate the inter-relationships between PIF and these constructs. This information can be used by medical educators when designing curricula and educational strategies intended to enhance PIF. Future work should seek to assess the influence of these constructs longitudinally.

## Data availability statement

The data analyzed in this study is subject to the following licenses/restrictions: The data that support the findings of this study are available from the corresponding author but restrictions apply to the availability of these data, which were used with permission for the current study, and so are not publicly available. Data is however available from the authors upon reasonable request and with permission of RCSI Research Ethics Committee. Requests to access these datasets should be directed to aineryan@rcsi.ie.

## Ethics statement

The studies involving humans were approved by the Royal College of Surgeons in Ireland Research Ethics Committee (REC202005016). The studies were conducted in accordance with the local legislation and institutional requirements. The participants provided their written informed consent to participate in this study.

## Author contributions

AR: Conceptualization, Data curation, Investigation, Methodology, Project administration, Resources, Visualization, Writing – original draft, Writing – review & editing. CM: Conceptualization, Investigation, Visualization, Writing – original draft, Writing – review & editing. DB: Conceptualization, Formal analysis, Methodology, Validation, Visualization, Writing – original draft, Writing – review & editing. AH: Conceptualization, Project administration, Resources, Supervision, Visualization, Writing – original draft, Writing – review & editing. DH: Project administration, Resources, Supervision, Visualization, Writing – original draft, Writing – review & editing. FB: Formal analysis, Methodology, Validation, Writing – original draft, Writing – review & editing. SG: Validation, Visualization, Writing – original draft, Writing – review & editing. AB: Validation, Visualization, Writing – original draft, Writing – review & editing. FD: Conceptualization, Investigation, Methodology, Project administration, Resources, Supervision, Validation, Visualization, Writing – original draft, Writing – review & editing.

## Footnotes

^1^ www.rcsi.com

## References

[1] Cruess R Cruess S Boudreau J Snell L Steinert Y. Reframing medical education to support professional identity formation. Acad Med. (2014) 89:1446–51. doi: 10.1097/ACM.0000000000000427, PMID: 25054423

[2] Jarvis-Selinger S Pratt DD Regehr G. Competency is not enough: integrating identity formation into the medical education discourse. Acad Med. (2012) 87:1185–90. doi: 10.1097/ACM.0b013e3182604968 22836834

[3] Frost HD Regehr G. "I am a doctor": negotiating the discourses of standardization and diversity in professional identity construction. Acad Med. (2013) 88:1570–7. doi: 10.1097/ACM.0b013e3182a34b05 23969361

[4] de Lasson L Just E Stegeager N Malling B. Professional identity formation in the transition from medical school to working life: a qualitative study of group-coaching courses for junior doctors. BMC Med Educ. (2016) 16:165. doi: 10.1186/s12909-016-0684-3, PMID: 27342973 PMC4919855

[5] Matthews J Bialocerkowski A Molineux M. Professional identity measures for student health professionals - a systematic review of psychometric properties. BMC Med Educ. (2019) 19:308. doi: 10.1186/s12909-019-1660-5, PMID: 31409410 PMC6693256

[6] Cruess SR Cruess RL. Teaching professionalism - why, what and how. Facts Views Vis Obgyn. (2012) 4:259–65. PMID: 24753918 PMC3987476

[7] Mount GR Kahlke R Melton J Varpio L. A critical review of professional identity formation interventions in medical education. Acad Med. (2022) 97:S96–s106. doi: 10.1097/ACM.0000000000004904, PMID: 35947478

[8] Kirk LM. Professionalism in medicine: definitions and considerations for teaching. Proc. (2007) 20:13–6. doi: 10.1080/08998280.2007.11928225, PMID: 17256035 PMC1769526

[9] Sternszus R Slattery NK Cruess RL ten Cate O Hamstra SJ Steinert Y. Contradictions and opportunities: reconciling professional identity formation and competency-based medical education. Perspect Med Educ. (2023) 12:507–16. doi: 10.5334/pme.1027 37954041 PMC10637293

[10] Goldie J. The formation of professional identity in medical students: considerations for educators. Med Teach. (2012) 34:e641–8. doi: 10.3109/0142159X.2012.687476 22905665

[11] Holden M Buck E Clark M Szauter K Trumble J. Professional identity formation in medical education: the convergence of multiple domains. HEC Forum. (2012) 24:245–55. doi: 10.1007/s10730-012-9197-6, PMID: 23104548

[12] Cruess RL Cruess SR Boudreau JD Snell L Steinert Y. A schematic representation of the professional identity formation and socialization of medical students and residents: a guide for medical educators. Acad Med. (2015) 90:718–25. doi: 10.1097/ACM.0000000000000700, PMID: 25785682

[13] Cohen MJ Kay A Youakim JM Balaicuis JM. Identity transformation in medical students. Am J Psychoanal. (2009) 69:43–52. doi: 10.1057/ajp.2008.38 19295620

[14] Vågan A. Medical students' perceptions of identity in communication skills training: a qualitative study. Med Educ. (2009) 43:254–9. doi: 10.1111/j.1365-2923.2008.03278.x, PMID: 19250352

[15] McGurgan P Calvert KL Narula K Celenza A Nathan EA Jorm C. Medical students’ opinions on professional behaviours: the professionalism of medical students’ (PoMS) study. Med Teach. (2020) 42:340–50. doi: 10.1080/0142159X.2019.1687862, PMID: 31738619

[16] Altirkawi K. Teaching professionalism in medicine: what, why and how? Sudan J Paediatr. (2014) 14:31–8. PMID: 27493387 PMC4949913

[17] Quince T Abbas M Murugesu S Crawley F Hyde S Wood D. Leadership and management in the undergraduate medical curriculum: a qualitative study of students’ attitudes and opinions at one UK medical school. BMJ Open. (2014) 4:e005353. doi: 10.1136/bmjopen-2014-005353, PMID: 24965917 PMC4078777

[18] Farquhar J Kamei R Vidyarthi A. Strategies for enhancing medical student resilience: student and faculty member perspectives. Int J Med Educ. (2018) 9:1–6. doi: 10.5116/ijme.5a46.1ccc, PMID: 29334480 PMC5834818

[19] Brown MEL Whybrow P Kirwan G Finn GM. Professional identity formation within longitudinal integrated clerkships: a scoping review. Med Educ. (2021) 55:912–24. doi: 10.1111/medu.14461, PMID: 33529395

[20] Chin D Phillips Y Woo M Clemans A Yeong P. Key components that contribute to professional identity development in internships for Singapore’s tertiary institutions: a systematic review. Asian J Scholars Teach Learn. (2020) 10:89–113.

[21] Sternszus R Boudreau JD Cruess RL Cruess SR Macdonald ME Steinert Y. Clinical Teachers' perceptions of their role in professional identity formation. Acad Med. (2020) 95:1594–9. doi: 10.1097/ACM.0000000000003369, PMID: 32271232

[22] Orsmond P McMillan H Zvauya R. It's how we practice that matters: professional identity formation and legitimate peripheral participation in medical students: a qualitative study. BMC Med Educ. (2022) 22:91. doi: 10.1186/s12909-022-03107-1, PMID: 35139839 PMC8830078

[23] Ryan A Hickey A Harkin D Boland F Collins ME Doyle F. Professional identity formation, professionalism, leadership and resilience (PILLAR) in medical students: methodology and early results. J Med Educat Curri Develop. (2023) 10:23821205231198921. doi: 10.1177/23821205231198921, PMID: 37692556 PMC10483968

[24] Weaver R Peters K Koch J Wilson I. 'Part of the team': professional identity and social exclusivity in medical students. Med Educ. (2011) 45:1220–9. doi: 10.1111/j.1365-2923.2011.04046.x, PMID: 21999250

[25] Maile E McKimm J Till A. Exploring medical leader identity and its formation. Leadersh Health Serv (Bradf Engl). (2019) 32:584–99. doi: 10.1108/LHS-12-2018-0066, PMID: 31612786

[26] Veen M Skelton J de la Croix A. Knowledge, skills and beetles: respecting the privacy of private experiences in medical education. Perspect Med Educ. (2020) 9:111–6. doi: 10.1007/S40037-020-00565-5, PMID: 32026318 PMC7138766

[27] Cruess S Cruess R Steinert Y. Supporting the development of a professional identity: general principles. Med Teach. (2019) 41:641–9. doi: 10.1080/0142159X.2018.1536260, PMID: 30739517

[28] Kalet A Buckvar-Keltz L Harnik V Monson V Hubbard S Crowe R. Measuring professional identity formation early in medical school. Med Teach. (2017) 39:255–61. doi: 10.1080/0142159X.2017.1270437 28033728

[29] Wald HS White J Reis SP Esquibel AY Anthony D. Grappling with complexity: medical students’ reflective writings about challenging patient encounters as a window into professional identity formation. Med Teach. (2019) 41:152–60. doi: 10.1080/0142159X.2018.1475727, PMID: 29944035

[30] Wald HS. Professional identity (trans)formation in medical education: reflection, relationship, resilience. Acad Med. (2015) 90:701–6. doi: 10.1097/ACM.0000000000000731, PMID: 25881651

[31] von Elm E Altman DG Egger M Pocock SJ Gøtzsche PC Vandenbroucke JP. The strengthening the reporting of observational studies in epidemiology (STROBE) statement: guidelines for reporting observational studies. Lancet. (2007) 370:1453–7. doi: 10.1016/S0140-6736(07)61602-X 18064739

[32] Crossley J Vivekananda-Schmidt P. The development and evaluation of a professional self identity questionnaire to measure evolving professional self-identity in health and social care students. Med Teach. (2009) 31:e603–7. doi: 10.3109/01421590903193547, PMID: 19995162

[33] Reddy ST Farnan JM Yoon JD Leo T Upadhyay GA Humphrey HJ. Third-year medical students' participation in and perceptions of unprofessional behaviors. Acad Med. (2007) 82:S35–9. doi: 10.1097/ACM.0b013e3181405e1c, PMID: 17895686

[34] Wilkinson TJ Wade WB Knock LD. A blueprint to assess professionalism: results of a systematic review. Acad Med. (2009) 84:551–8. doi: 10.1097/ACM.0b013e31819fbaa2, PMID: 19704185

[35] NHS Leadership Academy. Medical leadership competency framework: self-assessment tool. United Kingdom: NHS Institute for Innovation and Improvement (2012).

[36] Smith BW Dalen J Wiggins K Tooley E Christopher P Bernard J. The brief resilience scale: assessing the ability to bounce back. Int J Behav Med. (2008) 15:194–200. doi: 10.1080/10705500802222972, PMID: 18696313

[37] Team R Core. R: a language and environment for statistical computing [computer program]. Vienna: R Foundation for Statistical Computing (2013).

[38] Korkmaz S Goksuluk D Zararsiz G. MVN: multivariate normality tests. The R Journal. (2014) 6.

[39] Rosseel YJT Rockwood N Oberski D Byrnes J Vanbrabant L Savalei V. The Lavaan tutorial: department of data analysis. Belgium: Ghent University (2023).

[40] Revelle W. Procedures for personality and psychological research. Evanston, IL, Version = 1.7. 8. Available at: https://CRAN.R-project.org/package=psych: Northwestern University (2017).

[41] Jorgensen TD PS Schoemann AM Rosseel Y Miller P Quick C Garnier-Villarreal M. SemTools: useful tools for structural equation modeling. R package version 0.5-6. Available at: https://CRAN.R-project.org/package=semTools (2022).

[42] Kelley K. The MBESS r package MBESS. Available at: https://cranr-projectorg/web/packages/MBESS/MBESSpdf (2022).

[43] Cangur S Ercan I. Comparison of model fit indices used in structural equation modeling under multivariate normality comparison of model fit indices used in structural equation modeling under multivariate normality. J Modern Appl Statist Methods. (2015) 14:152–167. doi: 10.22237/jmasm/1430453580

[44] Lt H Bentler PM. Cutoff criteria for fit indexes in covariance structure analysis: conventional criteria versus new alternatives. Struct Equ Model Multidiscip J. (1999) 6:1–55. doi: 10.1080/10705519909540118

[45] Schermelleh-Engel K Moosbrugger H Müller H. Evaluating the fit of structural equation models: tests of significance and descriptive goodness-of-fit measures. Methods Psychol Res. (2003) 8:23–74.

[46] Schumacker RE. Learning statistics using R. London: (2015) Available at: https://methods.sagepub.com/book/learning-statistics-using-r.

[47] Alumran A Hou XY Sun J Yousef AA Hurst C. Assessing the construct validity and reliability of the parental perception on antibiotics (PAPA) scales. BMC Public Health. (2014) 14:73. doi: 10.1186/1471-2458-14-73, PMID: 24456730 PMC3909352

[48] Chen FF. Sensitivity of goodness of fit indexes to lack of measurement invariance. Struct Equ Model Multidiscip J. (2007) 14:464–504. doi: 10.1080/10705510701301834

[49] Fornell C Larcker DF. Evaluating structural equation models with unobservable variables and measurement error. J Mark Res. (1981) 18:39–50. doi: 10.1177/002224378101800104

[50] Archer R Elder W Hustedde C Milam A Joyce J. The theory of planned behaviour in medical education: a model for integrating professionalism training. Med Educ. (2008) 42:771–7. doi: 10.1111/j.1365-2923.2008.03130.x, PMID: 18715476

[51] Putnick DL Bornstein MH. Measurement invariance conventions and reporting: the state of the art and future directions for psychological research. Dev Rev. (2016) 41:71–90. doi: 10.1016/j.dr.2016.06.004, PMID: 27942093 PMC5145197

[52] Kalet A Buckvar-Keltz L Monson V Harnik V Hubbard S Crowe R. Professional identity formation in medical school: one measure reflects changes during pre-clerkship training. MedEdPublish. (2016) 7:41. doi: 10.15694/mep.2018.0000041.1 PMC10712001 38089226

[53] Jorgensen TD. Applying permutation tests and multivariate modification indices to Configurally invariant models that need Respecification. Front Psychol. (2017) 8:1455. doi: 10.3389/fpsyg.2017.01455, PMID: 28883805 PMC5573877

[54] Klemenc-Ketis Z Vrecko H. Development and validation of a professionalism assessment scale for medical students. Int J Med Educ. (2014) 5:205–11. doi: 10.5116/ijme.544b.7972, PMID: 25382090 PMC4249760

