# Supplementary material for: Do professionalism, leadership, and resilience combine for professional identity formation? Evidence from confirmatory factor analysis
Source: Front Med (Lausanne). 2024 Jun 13;11:1385489. doi: 10.3389/fmed.2024.1385489 (PMC11208471; doi:10.3389/fmed.2024.1385489)
Supplement: Supplementary file 1 [file Table_1.DOCX]

## Appendix 1: Data Screening and Assumptions Testing

Data were screened for univariate outliers using the criterion of 2.2 times the inter-quartile range (2.2xIQR) (45), with no outliers being found. Listwise deletion was use to remove missing values, resulting in a valid sample of n = 1234 and a sample to variable ratio of 27:1 (after rounding). Proposed acceptable sample to variable ratios within for conducting factor analysis social sciences vary widely, with ratios ranging from as low as 3:1 up to 10:1 (46). Based on the highest ratio reported by Williams et al., a ratio of 27:1 is indicative of an appropriate sample to conduct confirmatory factor analysis.

Univariate normality was examined using the Sharpiro-Wilk test, while the Henze-Zirkler test were uses to assess the multivariate normality of the data (47). The Shapiro-Wilk test produced a statistically significant test statistic for each item and the Henze-Zirkler test produces a statistically significant test statistic for the multivariate normality of all items (1.01, p < .001). The null hypothesis that data are normally distributed was rejected, with the results of these tests indicating a violation of multivariate normality. Descriptive statistics are presented in Table 1.
